# In Response to Errors in Antibiotic sensitivity testing: Barking up the wrong tree

**DOI:** 10.1016/j.amsu.2022.104112

**Published:** 2022-07-10

**Authors:** Hassan Mumtaz, Hassan ul Hussain, Shahzaib Ahmad

**Affiliations:** Public Health Scholar, Health Services Academy, Islamabad, Pakistan; Dow University of Health Sciences, 74200, Karachi, Pakistan; King Edward Medical University, Pakistan

Dear Editor

This is in response to a correspondence written “Errors in Antibiotic sensitivity testing: Barking up the wrong tree” [1] on my article published in Annals of Medicine & Surgery, titled “Twelve-year trend of Escherichia coli antibiotic resistance in the Islamabad population” [2].

Mr. Harit Kumar and Dr Nitin Kumar claim that Antibiotic susceptibility testing (AST) is a standardized procedure which need to be performed and interpreted as per the recommended guidelines like Clinical and Laboratory standards Institute (CLSI) or European Committee on Antimicrobial Susceptibility Testing (EUCAST). However, the authors in this study have not mentioned at all about any guidelines to conduct AST and interpretation of the result [3,4].

Antibiotic susceptibility testing (AST) was performed and interpreted as per the recommended guidelines from Clinical and Laboratory standards Institute (CLSI However, I acknowledge that it’s not mentioned in the study.

**Figure:**
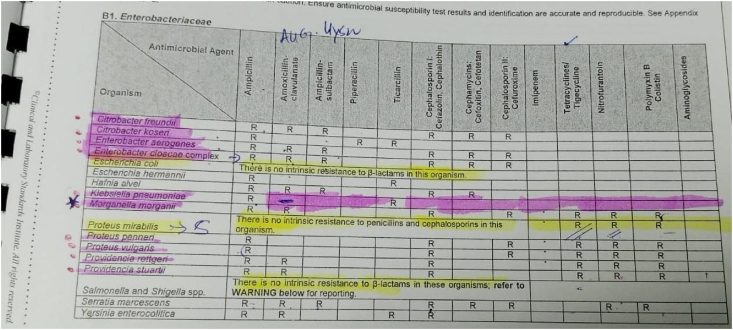

The Correspondence Authors made another claim that we have wrongly chosen and documented the concentrations of different antibiotics (Table 1) which were tested against the Uro-pathogenic E. coli strains. Moreover, the authors have just mentioned the name of antibiotic groups like Aminoglycosides and Cephalosporins but there are several antibiotics in each group which may not be tested using the same concentration. For instance, the potency of Gentamicin and Amikacin varies from each other [1].

**Table 1:** CLSI/EUCAST recommended potency of antibiotics.

S. No.	Antibiotics	Potency as mentioned in present study	Recommended potency by CLSI/EUCAST
1.	Amoxicillin/Clavulanic acid	625mg/1 gm	20/10 μg
2.	Trimethoprim/Sulphamethoxazole	160/800 mg	1.25/23.75 μg
3.	Aminoglycosides	80mg/500 mg	Gentamicin- 10 μg Amikacin- 30 μg
4.	Nitrofurantoin	3 gm	300 μg
5.	Cephalosporins	400 mg	30 μg
6.	Sulbactams	1 gm/2 gm	Not to be used alone
7.	Fosfomycin	100 mg	200 μg

The values mentioned in the antibiotic concentration portions of the methods [2] are the concentrations that were to be given to the patients for their treatment in specified doses normally available at the pharmacy, as decided by the Primary Care Specialist/Urologist according to his judgement & patient's body weight.

These concentrations were just mentioned in the study to let the readers & Clinicians know which antibiotics are prescribed routinely and the available doses in the consumer market [2].

Sulbactam, which is a beta-lactamase inhibitor and is recommended to be tested only in combination like Ampicillin-sulbactam etc., was tested alone and reported as 75% sensitive against Uro-pathogenic E. coli in 2021 in this study [1].

Sulbactam belongs to beta-lactamase inhibitor class and it was tested with Ampicillin and sulbactam. As well as sulbactam with Cephalosporins [2]. It's just a misunderstanding that authors have gone through.

There are different methods of performing antimicrobial susceptibility testing like diffusion method, dilution method, diffusion and dilution method etc. whereas the authors have not mentioned the name of method, opted in this study [5,6].

Diffusion method was chosen for our study [2].

In conclusion the antibiotic susceptibility test (AST) was conducted and the results evaluated. Both Ampicillin and Sulbactam were evaluated for their beta-lactamase inhibitor properties. The concentrations of these antibiotics were only disclosed so that readers and doctors could see what antibiotics are commonly administered. I think it's just an author's misunderstanding, we used the diffusion method.

## Ethical approval

Not applicable.

## Sources of funding

Not applicable.

## Author contribution

Dr Hassan Mumtaz, Dr Shahzaib Ahmed, Dr Hassan ul Hussain have equally contributed in writing the final manuscript.

## Registration of research studies

1. Name of the registry: NA
2. Unique Identifying number or registration ID: NA
3. Hyperlink to your specific registration (must be publicly accessible and will be checked): NA

## Guarantor

Not applicable.

## Consent

Not applicable.

## Declaration of competing interest

There is No conflict of interest.
